# A MRI-based radiomics nomogram for evaluation of renal function in ADPKD

**DOI:** 10.1007/s00261-022-03433-4

**Published:** 2022-02-13

**Authors:** Xiaojiao Li, Qingwei Liu, Jingxu Xu, Chencui Huang, Qianqian Hua, Haili Wang, Teng Ma, Zhaoqin Huang

**Affiliations:** 1 Department of Radiology, Shandong Provincial Hospital, Cheeloo College of Medicine, Shandong University, No.324, jingwuweiqi Road, Jinan, 250021 Shandong China; 2 Department of Research Collaboration, R&D Center, Beijing Deepwise & League of, PHD Technology Co.Ltd, Beijing, China

**Keywords:** Autosomal dominant polycystic key disease, radiomics nomogram, Evaluation of renal function, MRI

## Abstract

**Objectives:**

This study is aimed to establish a fusion model of radiomics-based nomogram to predict the renal function of autosomal dominant polycystic kidney disease (ADPKD).

**Methods:**

One hundred patients with ADPKD were randomly divided into training group (*n* = 69) and test group (*n* = 31). The radiomics features were extracted from T1-weighted fat suppression images (FS-T1WI) and T2-weighted fat suppression images (FS-T2WI). Decision tree algorithm was employed to build radiomics model to get radiomics signature. Then multivariate logistic regression analysis was used to establish the radiomics nomogram based on independent clinical factors, conventional MR imaging variables and radiomics signature. The receiver operating characteristic (ROC) analysis and Delong test were used to compare the performance of radiomics model and radiomics nomogram model, and the decision curve to evaluate the clinical application value of radiomics nomogram model in the evaluation of renal function in patients with ADPKD.

**Results:**

Fourteen radiomics features were selected to establish radiomics model. Based on FS-T1WI and FS-T2WI sequences, the radiomics model showed good discrimination ability in training group and test group [training group: (AUC) = 0.7542, test group (AUC) = 0.7417]. The performance of radiomics nomogram model was significantly better than that of radiomics model in all data sets [radiomics model (AUC) = 0.7505, radiomics nomogram model (AUC) = 0.8435, *p* value = 0.005]. The analysis of calibration curve and decision curve showed that radiomics nomogram model had more clinical application value.

**Conclusion:**

radiomics analysis of MRI can be used for the preliminary evaluation and prediction of renal function in patients with ADPKD. The radiomics nomogram model shows better prediction effect in renal function evaluation, and can be used as a non-invasive renal function prediction tool to assist clinical decision-making.

**Trial Registration:**

ChiCTR, ChiCTR2100046739. Registered 27 May 2021—retrospectively registered, http://www.ChiCTR.org.cn/showproj.aspx?proj=125955.

**Graphical abstract:**

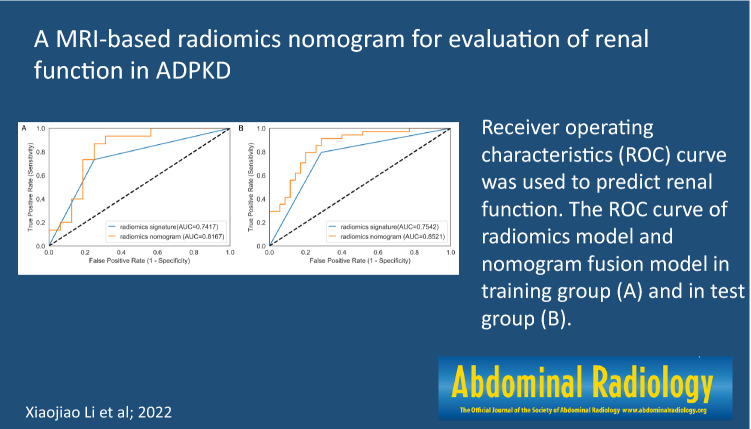

## Introduction

Autosomal dominant polycystic kidney disease (ADPKD) is the most common hereditary kidney disease, which main manifestation is the formation of many fluid filled cysts in the kidney. The cysts gradually increase and eventually damage the normal renal structure and function, leading to end-stage renal disease (ESRD) [1, 2]. ADPKD has become the fourth major cause of ESRD. According to guideline 3 issued by KDOQI [3], chronic kidney disease (CKD) is divided into five stages. When the disease develops to CKD3 or above (GFR < 60 ml/min per 1.73 m^2^), the risk of complications and progression to ESRD is significantly increased. Therefore, it is very important to evaluate and monitor renal function of ADPKD patients. At present, estimated glomerular filtration rate (eGFR) is the most commonly used laboratory index to monitor renal function in clinical practice since is easier to obtain than inulin clearance rate or 99 mTc-DTPA renal scan. However, the early renal function changes are not easily reflected by GFR due to the existence of compensatory renal ultrafiltration [4, 5]. Finding a non-invasive method to evaluate the renal function of ADPKD patients is the problem to be solved in this study.

Monitoring over time the total kidney volume (TKV) and height-adjusted TKV (ht-TKV) by MRI represent a non-invasive and non-radiation method to assess the risk of progression to ESRD of ADPKD patients [6, 7]. However, renal volume does not always predict changes in renal function, for example, in patients with few large cysts or renal atrophy caused by ischemia or urinary tract obstruction [3]. In recent years, radiomics has attracted more and more attention in staging or prediction of various organ diseases, which can achieve the purpose of evaluating disease progression, prognosis and predicting treatment response through feature extraction of image big data and transforming medical data into high-dimensional data [9, 10]. radiomics research applied to renal diseases mainly focuses on renal neoplastic diseases, such as distinguishing the histological grade of renal cell carcinoma, predicting synchronous distant metastasis of renal clear cell carcinoma, predicting preoperative staging and scoring of renal clear cell carcinoma, etc [11–13]. However, there are few reports on radiomics of renal non-neoplastic lesions, especially for patients with ADPKD. Kline et al. [14] conducted a preliminary study on ADPKD, and showed that T2-weighted image texture feature analysis can predict the decline of renal function. At present, there is no report about characteristics extraction of renal MR multi-sequence images of ADPKD patients combined with clinical factors to predict the disease progression.

In this study, the radiomics features of FS-T1WI and FS-T2WI sequence images of patients with ADPKD were extracted to evaluate and predict the progress of polycystic kidney disease, and to further establish and verify a new radiomics nomograms model to predict the progress of polycystic kidney disease by combining multiple clinical factors and conventional MR imaging variables.

## Material and methods

### Research methods

A total of 100 patients with ADPKD in our hospital were retrospectively analyzed from January 2017 to December 2019, including 48 males and 52 females, range 24–76 year, average age 49.11 ± 9.85. All the patient specimens with their families have been informed and signed the informed consent. Meanwhile, the study was approved by the ethics committee of our hospital (registration No.: ChiCTR2100046739).

The patients were randomly divided into two groups, 69 in the training group and 31 in the test group for prediction model training and test. According to the grading standard of chronic kidney disease issued by KDOQI, the patients were divided into two groups. There were 51 cases with glomerular filtration rate (GFR) ≥ 60 ml/min per 1.73 m^2^ (including CKD1 and CKD2), and 49 cases with GFR < 60 ml/min per 1.73 m^2^ (above CKD3).

### Inclusive criteria

All patients underwent routine MRI including FS-T1WI and FS-T2WI before treatment. There is no artifact that affects the image radiomics analysis. All laboratory tests should be performed before treatment, and the interval between MRI and laboratory tests should not exceed 72 h.

### Exclusion criteria

Contraindications of MRI examination. The boundary between renal lesions and surrounding structures is unclear.

The clinical data and MR data included sex, hypertension, albuminuria (grade 0–3), estimate total kidney volume (eTKV), maximum cyst diameter, hemorrhage cyst diameter, urine leukocyte and urine erythrocyte. The eTKV was estimated using the formula of an ellipsoid (eTKV = π/6 × length × width × depth).

Two doctors with 10-year and 8-year experience in imaging diagnosis were involved in the joint interpretation. Through the comprehensive observation of transverse, sagittal and coronal images, the largest cyst was determined and the length of the largest section was selected. The largest bleeding cyst was the largest cyst with short T1 and short T2 signal.

### Examination method

All patients underwent routine renal MRI scan (3.0 T Siemens MRI scanner) in supine position and head advanced position. The signals were received by 8-channel abdominal phased array coil (Magnetom Verio, Siemens, Erlangen, Germany). The scanning scope covered all kidneys.

Before scanning, the patients were forbidden to eat for 6–8 h. The FSE sequence of respiratory triggered fat suppression was used in transverse T2WI. Scanning parameters: FS-T2WI: TR 2000 ~ 6000 ms, TE 80 ~ 104 ms, echo chain length 8–16, matrix 320 × 224, layer thickness of 6 mm, layer spacing of 0.6 mm, field of vision of 36 cm × 36 cm ~ 40 cm o chai; FS-T1WI: TR 3.92 ms, TE 1.39 ms, echo chain length 8–16, matrix 320 × 224, layer thickness of 3–5 mm, layer spacing of 1 mm.

### Image analysis

In this study, radiomics analysis was performed in the Dr. Wise Multimodal Research Platform, Beijing Deepwise & League of PHD Technology Co., Ltd, Beijing, China, including image annotation, feature extraction, selection and modeling.

### Image segmentation

The FS-T1WI and FS-T2WI mages of all patients were exported in "*. DICOM" format in picture archiving and communication system (PACS) and uploaded to Dr. Wise Multimodal Research Platform. The software operation and images interpretation were operated by two doctors with 10 and 8 years of experience in imaging diagnosis. Regarding the inconsistent opinions, the two doctors reached an agreement through discussions.

### Methods of image delineating

Firstly, the kidney was delineated as a whole. Secondly, the volume of interest (VOI) of the kidney was obtained by ROI delineation on all levels of the kidney contour except for the two levels where the lesion just appeared and was about to disappear. Thirdly, semi-automatic delineation was used, and the delineation software was built in the radiomics platform, which could automatically identify the rough contour of lesions. Manual adjustment was adopted for those with unsatisfactory delineation effect. Sagittal and coronal images can be combined for localization when necessary.

### Feature extraction

For images with different resolutions, B-spline interpolation sampling technology was used for resampling. After resampling, all images had the same resolution [1, 1, 1] to achieve the same image resolution. At the same time, for the original image and the transformed image, the radiomics features of the original image and the transformed image were extracted, including first-order features, shape features, gray level co-occurrence matrix (GLCM), gray level run length matrix (GLRLM), gray level size matrix (GLSZM), and gray level dependence matrix (GLDM). A total of 1648 radiomics features were extracted, including 14 shape features, 342 first-order features and 1292 texture features. Feature extraction was performed on two sequences respectively. A total of 1648 radiomics features were extracted on each sequences images, including 14 shape features, 342 first-order features and 1292 texture features. Feature extraction was performed on two sequences respectively.

### radiomics feature selection

In order to eliminate the scale difference of image radiomics features, feature standardization was carried out before feature selection. All features were Z-score normalized (subtracted the mean and divided by the standard deviation). At the same time, in order to eliminate the impact of different Sketchers, the consistency of features extracted by different Sketchers was analyzed, including the annotation of the same doctor at different time points and the annotation of different doctors. Features with consistency less than 0.75 was not be included. After the completion of the consistency analysis, further analysis of variance (ANOVA) was used to screen out the relevant features of polycystic kidney function assessment. Finally, least absolute shrinkage and selection operator (Lasso) were used for feature final selection. In this process, the coefficients of most of the features would become zero, and no zero features would be used for modeling.

### radiomics modeling and nomogram modeling

After feature selection, decision tree machine learning algorithm was used to build the radiomics model. Decision tree was a machine learning algorithm commonly used in binary classification problem. In this study, 69 data sets were used as training sets, and 31 data sets were used as independent test sets to verify the performance of the model. In order to get the final radiomics signature, the features of the two sequences were fused to establish a decision tree model, and the final radiomics signature was obtained through the decision tree model. radiomics signature combined with clinical factors would be screen out by logistic regression univariate analysis and multivariate analysis to build radiomics nomogram model. The independent clinical factors related to polycystic kidney function were modeled by logistic regression, and finally nomogram model was obtained to evaluate renal function.

### Model evaluation and statistical methods

All statistical analyses were performed on R software (version 3.6.2, R Foundation for Statistical Computing, Vienna, Austria). Continuous variables were reported as means ± standard deviations (SD), and categorical variables were shown as numbers, as appropriate. The intra-class correlation coefficient (ICC) was used to evaluate the inter-observer and intra-observer agreement. ANOVA was used to select the relevant features for the assessment of polycystic kidney function. Categorical variables were compared by using the Chi-square test or Fisher exact test, and continuous variables were compared by using the Student’s *t* test or Mann–Whitney *U* test, as appropriate. Logistic regression univariate analysis and multivariate analysis were used to screen clinical risk factors. ROC analysis was conducted to evaluate the performance of each model, and the AUCs between different models were compared by Delong’s test. *P* values < 0.05 was considered statistically significant.

## Results

### Patient characteristics

As showed in Table 1, for albuminuria (*p* = 0.009), eTKV (*p* = 0.000) and maximum cyst diameter (*p* = 0.015), there were significant differences in training cohort. In test cohort, two characteristics existed significantly difference, including albuminuria (*p* = 0.000) and maximum cyst diameter (*p* = 0.004).

**Table 1:** Characteristics and MR imaging features in patients with ADPKD

Characteristics	Training cohort *N* = 69			Test cohort *N* = 31		
	GFR ≥ 60(*n* = 35)	GFR < 60(*n* = 34)	*p*	GFR ≥ 60(*n* = 16)	GFR < 60(*n* = 15)	*p*
Sex (Male/Female)	17/18	14/20	0.537	8/8	9/6	0.273
Hypertension (Present /Absent)	30/5	30/4	1.000	8/8	11/4	0.722
Albuminuria, (0 − + + +)	24/9/2/0	12/10/9/3	0.009**	13/1/1/1	2/5/7/1	0.000**
eTKV, (cm^3^)	1360.242 ± 852.910	2652.501 ± 1835.094	0.000**	1599.187 ± 1512.669	2682.949 ± 1639.780	0.745
Urine leukocyte	4.060 ± 8.324	12.738 ± 36.346	0.249	17.250 ± 48.563	4.720 ± 7.055	0.314
Urine erythrocyte	38.920 ± 207.304	2.318 ± 2.271	0.536	266.606 ± 977.066	47.960 ± 173.060	0.399
Maximum cyst diameter (cm)	4.779 ± 1.742	5.647 ± 1.632	0.015*	5.312 ± 2.357	5.982 ± 1.077	0.004**
Hemorrhage cyst diameter (cm)	2.505 ± 1.894	3.092 ± 1.424	0.057	2.081 ± 1.718	3.501 ± 1.192	0.333

* *p*<0.05; ** *p*<0.01

### Features selection and modeling

**Fig. 1:**
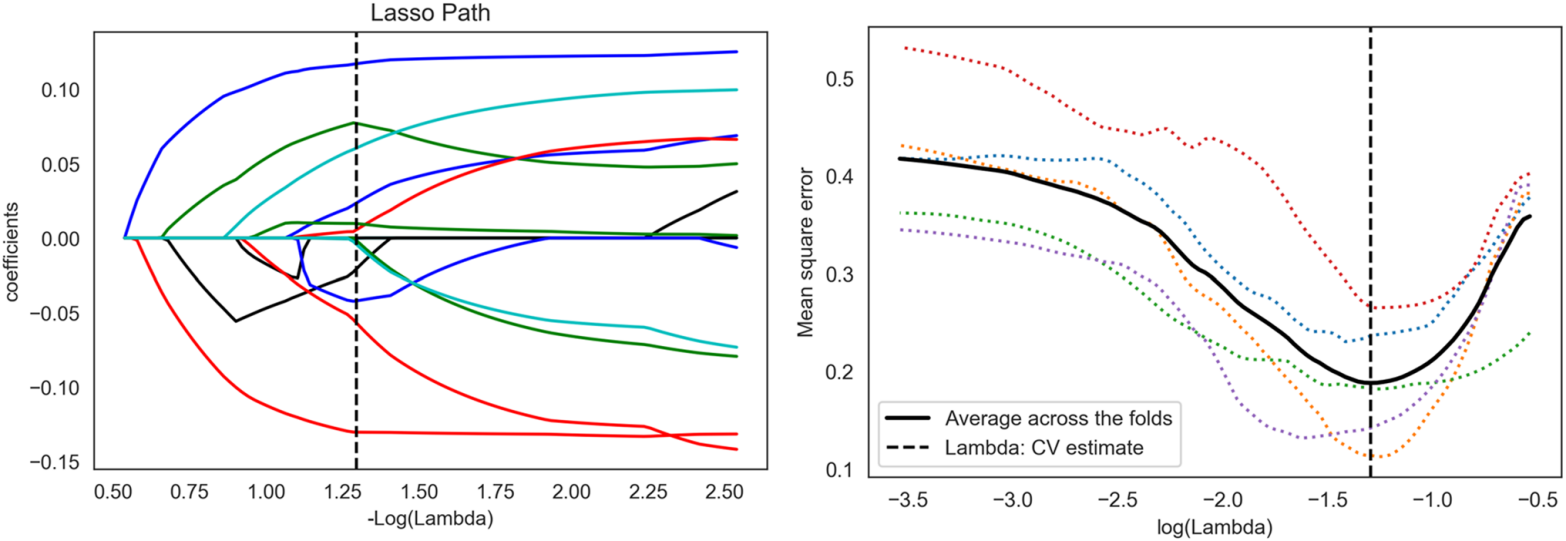
Lasso screening process of joint features. The best lambda value is 0.05062, − log (alpha) is 1.29564

**Fig. 2:**
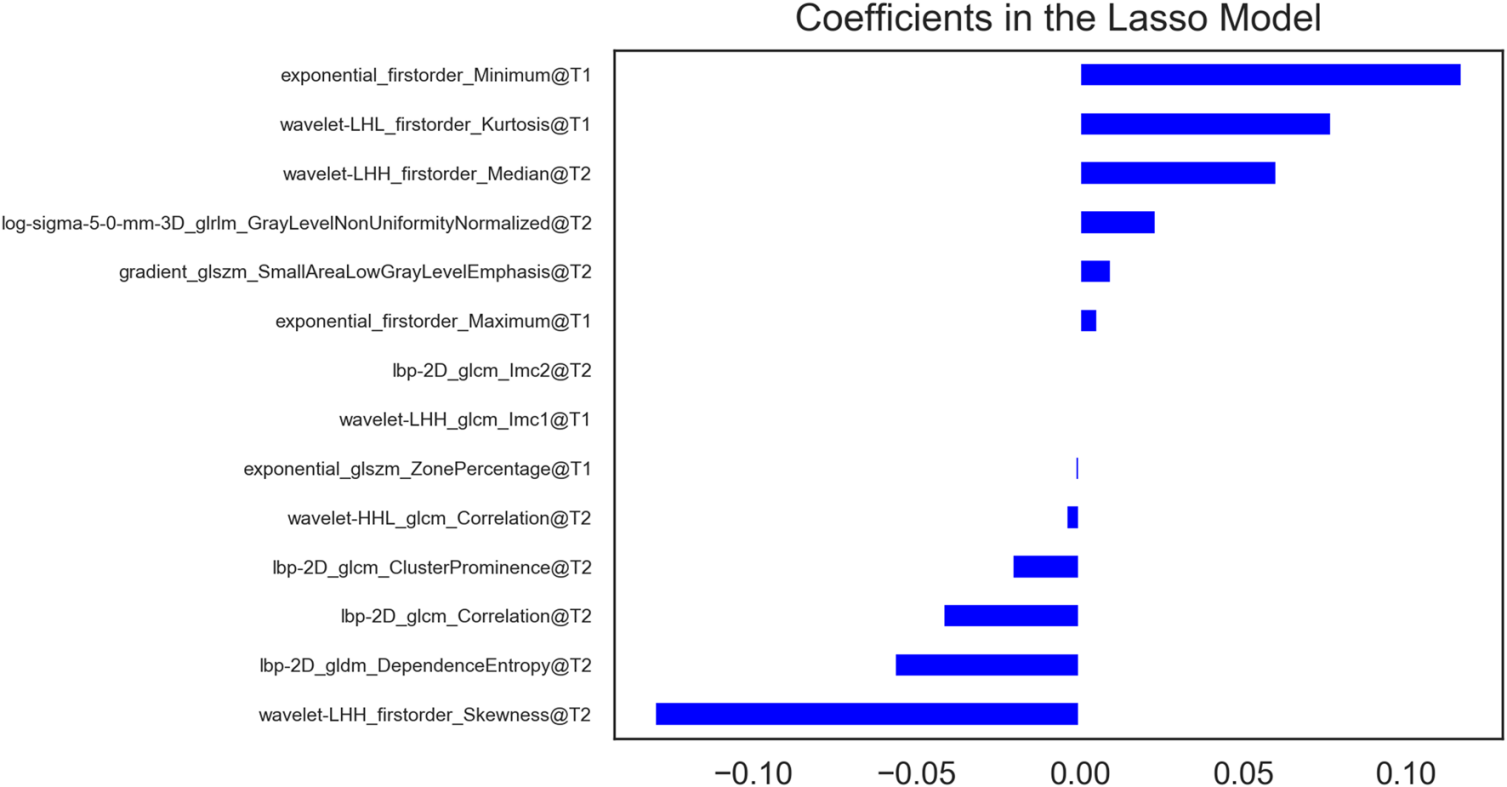
The 14 radiomics features and their weights screened by Lasso

For the features extracted from T1 sequence, 1358 robust features, including 6 shape features, 297 first-order features and 1055 texture features, were obtained after Inter- and intra-class correlation coefficients (ICCs) analysis. For T2 sequence, after ICCs analysis, 1597 features with consistency greater than 0.75 were retained. In order to combine T1 and T2 sequences for modeling, the features of the two sequences were combined to obtain the joint features of the two sequences for subsequent feature selection and modeling. Through one-way ANOVA analysis, a total of 1412 features were found to be related to renal function evaluation. Then, the features with the most diagnostic value were selected by Lasso (Fig. 1). Finally, 14 features were used for the final modeling, which included 5 radiomics features of T1 sequences and 9 radiomics features of T2 sequences. In terms of feature types, it included 5 first-order features and 9 texture features. The weights graph of features used for modeling is showed in Fig. 2. The decision tree model was built by using the final 14 features. The radiomics model showed good prediction effect in predicting the renal function of polycystic kidney. The AUC of training cohort was 0.7542 and the AUC of the test cohort was 0.7417.

### nomogram model

The combined sequence was modeled to obtain the final radiomics signature, and the final radiomics signature was used as an independent predictor that combined with clinical factors (gender, hypertension, urinary albumin, eTKV, urinary white blood cells, urinary red blood cells, maximum cyst length, maximum bleeding cyst length) to screen clinical variables by using logistic regression univariate analysis. The results of logistic regression including single factor analysis, multivariate analysis are showed in Table 2.

**Table 2:** Results of logistic regression

	Univariate analysis		Multivariate analysis		OR
	OR	*p* value	OR	*p* value	
Sex	5.3552	0.5374	NA	NA	
Hypertension	0.7188	0.7562	NA	NA	
Urinary red blood cells	0.055	0.3193	NA	NA	
Urinary white blood cells	0.0211	0.2789	NA	NA	
Urinary albumin	0.3501	0.0018	2.1229	0.0497	2.1229
Maximum cyst length	0.1516	0.0413	1.2055	0.7313	1.2055
Maximum bleeding cyst length	0.1497	0.1525	NA	NA	
eTKV	0.0003	0.0035	2.312	0.0745	2.312
radiomics	0.5656	< 0.0001	5.3552	0.0073	5.3552

**Fig. 3:**
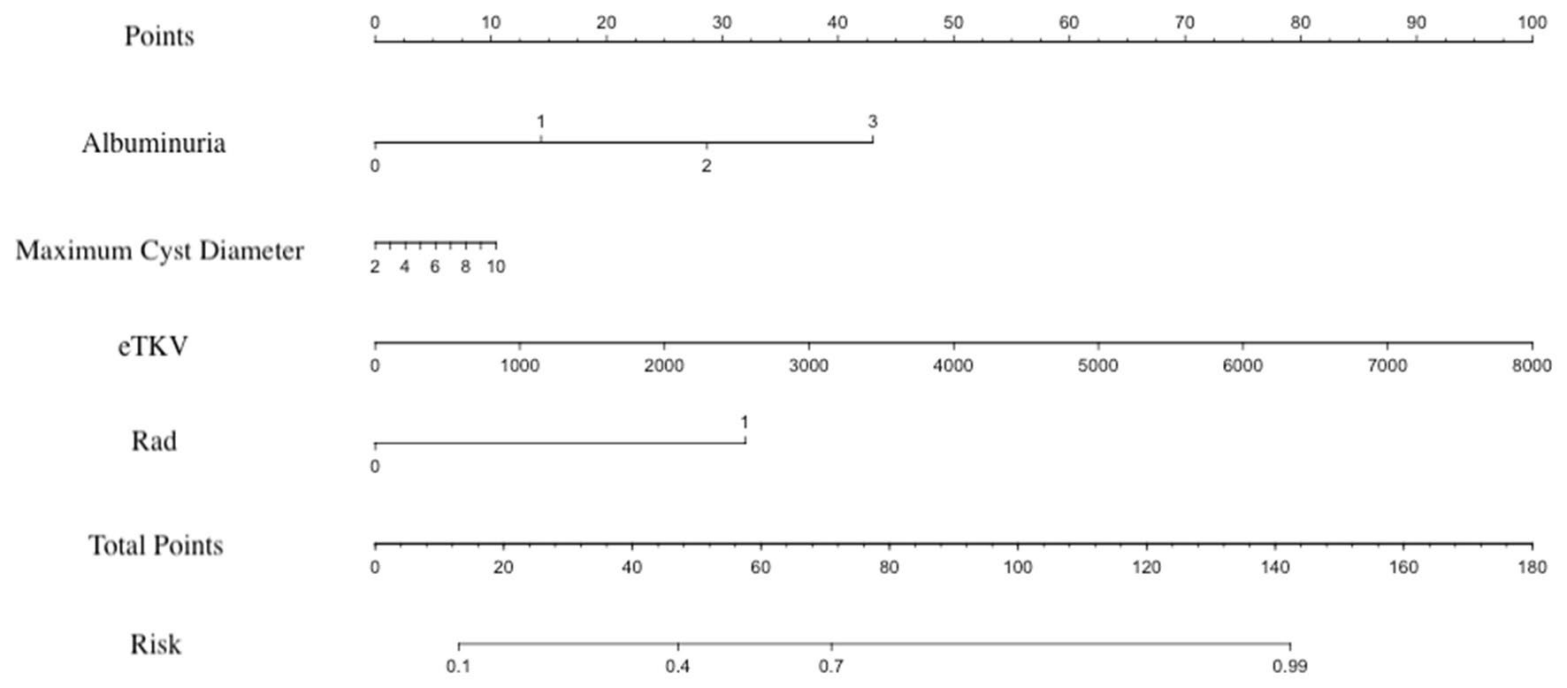
Visualization graph of nomogram. In the training cohort, the nomogram model was developed to predict the progression of renal function by combining urinary albumin, maximum cyst length, eKTV and radiomics

**Fig. 4:**
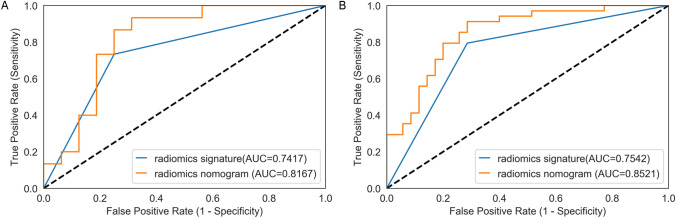
Receiver operating characteristics (ROC) curve was used to predict renal function. **A** The ROC curve of radiomics model and nomogram fusion model in training group. **B** The ROC curve of radiomics model and nomogram fusion model in test group

Finally, urinary albumin, maximum cyst diameter, eTKV and radiomics signature (Rad) were included to build the radiomics nomogram model (Fig. 3) by using logistic regression. In the multivariate model, radiomics signature was an independent risk factor. The AUC of nomogram model was 0.8521 in training group and 0.8167 in test group. The results of each model are displayed in Table 3. The ROC curves of radiomics model and nomogram model in the training group and test group are shown in Fig. 4.

**Table 3:** radiomics model and nomogram model

Model	Training				Test				All			
	AUC	SEN	SPE	ACC	AUC	SEN	SPE	ACC	AUC	SEN	SPE	ACC
radiomics model	0.7542	0.7143	0.7941	0.7536	0.7417	0.7500	0.7333	0.7419	0.7505	0.7255	0.7755	0.7500
nomogram model	0.8521	0.7143	0.9118	0.8116	0.8167	0.6875	0.9333	0.8065	0.8435	0.7059	0.8980	0.8000
*p*									0.005372

It can be seen from Table 3 and Fig. 4 that the nomogram model performs well in both the training group and the test group. The AUC of the nomogram model was higher than that of the radiomics model. In the whole data, nomogram model was significantly better than radiomics model (*p* = 0.005372).

**Fig. 5:**
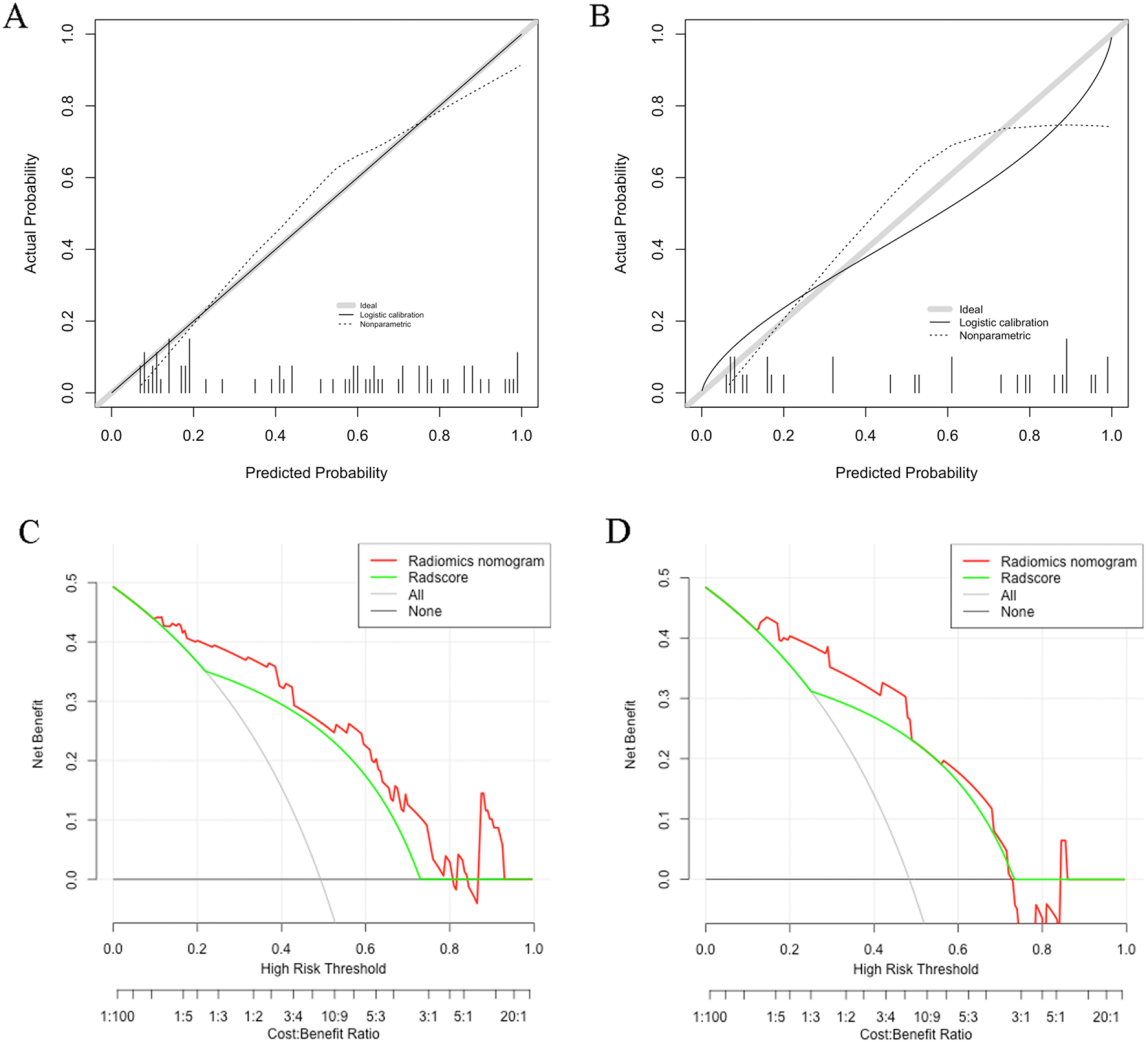
Calibration curve represents the goodness of fit prediction model, in which the 45 °C gray line represents the ideal prediction, and the solid line represents the prediction performance of nomogram model. The closer the real line is to the ideal prediction line, the better the prediction effect of nomogram model is. **A** Calibration curve in training group. **B** Calibration curve in test group. **C** Decision curve in training group. **D** Decision curve in test group. The y-axis indicates the net benefit; x-axis indicates threshold probability. The red line and green line represent net benefit of the nomogram and the radiomics signature, respectively. The gray line shows that GFR of all patients was less than 60 ml/min per 1.73 m^2^; The black line indicates that no patient is supposed to have GFR < 60 ml/min per 1.73 m^2^. nomogram model shows that the net benefits of training (**C**) and test (**D**) queues are optimal in most range of threshold probability

The calibration curves of nomogram model in training group and test group are shown in Figs. 5A, B. The nomogram model has satisfactory fitting degree based on the training set (Hosmer–Lemeshow test, *p* = 0.4058) and the test set (Hosmer–Lemeshow test, *p* = 0.2198). It showed that there was no statistical difference between the current nomogram model and the ideal perfect model, which was acceptable. The decision curves (Fig. 5C, D) of radiomics model (Radscore) and nomogram model showed that the clinical benefits of the nomogram model were greater than the radiomics model.

## Discussion

In this study, we developed and validated a combined radiomics model based on T1 and T2. A total of 14 features were selected, and the radiomics model has good predictive performance in the assessment of polycystic kidney function. In addition, multiple clinical data, conventional MR imaging variables and radiomics signature were integrated into a combined nomograms model, and the results showed that the combined nomograms model had the best prediction value in the whole group, with AUC of 0.8435.

After analyzing the observational data from five different sources, PKDOC (Association for improving prognosis of polycystic kidney disease) concluded that TKV was the most important index to predict the decline of eGFR [5]. Therefore, the TKV was combined with patient age and estimated glomerular filtration rate (eGFR) as a prognostic imaging biomarker for ADPKD research and clinical trials in the US Food and Drug Administration and the European Drug Administration. In CRISPII, height-adjusted baseline TKV (ht-TKV) was associated with CKD stage 3 development after 8 years of follow-up. However, GFR and TKV also have their limitations. Due to the existence of renal ultrafiltration, early renal function changes are not easily reflected by GFR, and GFR detection also has certain trauma. In addition, renal volume does not always predict changes in renal function, for example, in patients with few large cysts or renal atrophy due to ischemia or urinary tract obstruction [8]. The current Mayo imaging classification (MIC) [7] is only applicable to patients with typical diffuse cystic disease (Category 1), while for the remaining 5–10% of patients with atypical morphology (Category 2), the prediction of eGFR decline is poor, and there is still no applicable imaging-based risk model. Bae kt et al. [16] considered that the use of recalculated ht-TKV measurements excluding significant exogenic cysts would help to include category 2 patients and reclassify category 1 patients in the Mayo classification model. In recent years, radiomics has been widely used in a variety of organs and diseases, which can diagnose, treat and predict the prognosis of diseases. It is a new non-invasive method to assist clinical decision-making. A preliminary study on ADPKD showed that kidneys with random cysts have high entropy. T2WI texture features can predict the decline of renal function. These texture features indicate the degree of renal structural disorder, the difference in the size and number of cysts, and whether the kidney is composed of many similar regions [14]. In this study, we will further extract multi-sequence images to provide radiomics features, combined with clinical factors and conventional MR imaging variables for analysis.

Some studies have shown that the evaluation of spatial heterogeneity by texture analysis is best reflected by the whole tumor analysis [17–19]. ROI of T1 and T2 images can provide more information by selecting the whole kidney as a whole. T2-weighted images have higher soft tissue contrast and high intensity of renal cyst, which is easy to show the boundary between kidney and background tissue [20]. T1-weighted imaging can well show the structure of renal parenchyma, especially hemorrhagic cyst and residual renal parenchyma [21, 22]. Studies have shown that hemorrhagic cysts can lead to renal TKV increase, and has a good correlation with ht-TKV. The number of hemorrhagic renal cysts is a very useful feature to predict eEGFR [23]. In the process of radiomics feature extraction, we also found that T1 and T2 sequences provided different feature information. After all features were screened, there were 5 features based on T1 sequence and 9 features based on T2 sequence. The results showed that radiomics model could get better ROC curve in predicting renal function of ADPKD.

In nomogram model, radiomics signature (Radscore), multiple clinical data and conventional MR imaging variables were included, and the highest OR value of variable was 5.3552, which indicated that radiomics signature was strongly correlated with ADPKD renal function prediction [24]. The continuous increase of renal volume comes from the increasing number and size of cysts. Some studies have shown that large renal cysts (diameter ≥ 5 cm) are more harmful because they hinder the flow of blood and urine in a wide range of nephrons [25]. In the clinical treatment of ADPKD, surgery is often used to reduce the pressure of cyst on renal parenchyma, especially the pressure of large cyst on renal parenchyma [26–28]. nomogram fusion model also confirmed that the maximum cyst diameter and TKV could be used as independent predictors.

When the permeability of glomerular capillary wall increases, macromolecules, such as albumin, will infiltrate into the renal capsule cavity, resulting in proteinuria. Proteinuria is a clinical marker of chronic kidney disease and an effective predictor of disease progression. CRISP pointed out that elevated ht-TKV was associated with frequent and severe complications of ADPKD, such as hypertension, hematuria and proteinuria, and progressive loss of renal function [15, 29]. Previous studies in children and adults have shown that urinary protein and albumin are associated with faster progression of some more severe kidney diseases [30]. Another study has shown that hypertension and proteinuria are the main treatable risk factors for CKD progression in ADPKD patients [31]. Gansevoort et al. [32] found that the increase albuminuria was associated with rapid loss of eGFR in ADPKD patients. Compared with placebo, tolvaptan reduced albuminuria, which was not associated with blood pressure. The monitoring of urinary albumin can prompt whether drug intervention is needed to reduce urinary albumin and delay disease progression. Meanwhile, nomogram fusion model also proves that urinary albumin can be used as an independent predictor of the model. Finally, we come to the conclusion that the combination of clinical factors and radiomics model provides a new idea and method for the prediction of renal function progression in ADPKD. This method can be performed by means of conventional magnetic resonance imaging and clinical laboratory data. It is economical, effective and simple, and can not only effectively make up for the deficiency of GFR in the diagnosis of early renal dysfunction, but also serve as a routine and effective follow-up method to monitor the renal function of ADPKD patients.

The disadvantage of this study is that the total number of cases is small, and there is no multi-center data to verify. In addition, some clinical factors that could not be included in the analysis, such as the time point of hypertension measurement and long-term drug intervention.

## Conclusion

In conclusion, our research on radiomics analysis of MR images of patients with ADPKD shows that T1 and T2 feature extraction can obtain effective information for the evaluation of renal function, and clinical data fusion modeling can be used to evaluate the progress of renal function in patients with ADPKD. At the same time, it is proved that the nomogram model combined with clinical features and MR imaging variables can improve the diagnostic efficiency of simple radiomics model in a certain extent. The radiomics model and nomogram model can provide a new non-invasive method for evaluating renal function progress and clinical decision-making in patients with ADPKD. The results of this study need to be confirmed in prospective randomized multi-center clinical trials with a greater sample size in order to better describe the role of this MRI-based nomogram in follow-up of ADPKD patients.

## Data Availability

The datasets during and/or analyzed during the current study available from the corresponding author on reasonable request.
